# Sheath Formation in a Plasma with Regularized Kappa Distribution

**DOI:** 10.3390/e28020142

**Published:** 2026-01-27

**Authors:** Rui Huo

**Affiliations:** 1 Department of Physics, Taiyuan Normal University, Jinzhong 030619, China; 1022210006@tju.edu.cn; 2 Institute of Computational and Applied Physics, Taiyuan Normal University, Jinzhong 030619, China

**Keywords:** non-maxell velocity distribution, debye shielding, electrostatic sheath

## Abstract

Debye shielding in an electron–ion plasma with regularized kappa distribution is examined. An unmagnetized collisionless plasma sheath with regularized kappa distributed electrons is investigated and the modified Bohm criterion is derived. It is found that the variation of the electrostatic potential depends significantly on the superthermal index *κ* and cutoff parameter *α*. If *κ* < 3/2, a plasma sheath with a regularized kappa distribution exists. Our present work may be useful in understanding plasma processing and plasma sheaths in related plasma regions (i.e., Earth’s inner magnetosphere).

## 1. Introduction

In recent years, plasma sheaths have been one of the most researched topics in plasma physics owing to their unique characteristics. When plasma interacts with a solid wall, due to the electrons’ higher mobility than ions, there exists a non-neutral transition region between the plasma and the wall. The wall will repel the electrons and attract ions. Finally, a very narrow (a few electrons Debye length, *λ_De_*) positive space charge layer is formed, which is called the plasma sheath [1]. The properties of the plasma sheath are significantly different from those of the plasma body because of the presence of the electric field, and are extensively applied in many processes such as plasma probe measurements, plasma material surface modification, and fusion research [2,3,4].

Langmuir and Tonks first proposed the term “plasma sheath” in the 1920s [5]. Later, Bohm theoretically studied the necessary conditions for the existence of a steady-state sheath by using the fluid model [6]. It was concluded that the velocity of ions entering the edge of the plasma sheath must be greater than the ion acoustic velocity, which is known as the Bohm sheath criterion. After Bohm, many researchers found that the Bohm sheath criterion in an ion–electron plasma is modified by many factors, such as ion–electron collision, ion temperature, external magnetic field, so on. Liu et al. investigated the Bohm sheath criterion with a two-fluid model by considering collisions [7]. Hatami and Shokri studied the Bohm criterion in a collisional magnetized plasma with thermal ions [8]. The Bohm sheath criterion in multi-component plasma has been extensively discussed [9,10,11,12].

The works mentioned above studied the Bohm sheath criterion in the framework of Boltzmann–Gibbs statistics. However, in systems with long-range interactions such as plasma, particle distribution will deviate from the Maxwellian distribution and often exhibit a long power-law tail [13,14]. These high-energy particles are described appropriately by the kappa distribution proposed by Vasyliunas in 1968 [15]. Subsequently, people found that the application of the kappa distribution helps us to understand plasma environments better than the Maxwellian distribution, and employed it in space, solar, and other astrophysical plasma [16,17,18,19,20]. In spite of a wide range of applications in plasma, there exists a mathematical issue in the kappa distribution that the spectral index *κ* must satisfy *κ* > (*l* + 1)/2 where *l* is the order of moments. But the observations display the existence of suprathermal tails which correspond to lower *κ*. In order to avoid diverging moments, the regularized kappa distribution was introduced, with which arbitrary velocity moments are valid for all *κ* > 0 [21,22]. Recently, some authors have applied the regularized kappa distribution to investigate the wave and instability in space plasma. Liu et al. studied the dispersion and damping rate of Langmuir waves with regularized kappa distributed electrons. They found that in the region *κ* < 1.5, the damping rate of LW will be much larger than that with Maxwellian distributed electrons [23]. The effect of a regularized kappa distribution on various ion acoustic waves has also been discussed [24,25,26,27]. However, no one has applied the regularized kappa distribution to the sheath theory. The application of non-Maxwellian distributions in sheath theory has been quite extensive [28,29,30,31,32,33]. Therefore, the present work will be devoted to studying the effect of regularized kappa distribution on Debye shielding and the electrostatic sheath.

The paper is organized as follows. In Section 2, the Debye shielding in an electron–ion plasma with regularized kappa distribution is examined. In Section 3, the plasma sheath with regularized kappa distributed electrons is investigated. And in Section 4, the conclusion is given.

## 2. Debye Shielding

We are considering an unmagnetized collisionless ion–electron plasma with a regularized kappa distribution. The charge neutrality condition at equilibrium is *n_e_*_0_ = *n_i_*_0_ = *n*_0_. The high-energy electrons are modeled by the regularized kappa distribution with an electrostatic potential *ϕ* given as [21]

(1) $$f_{e}\left(v\right)=\frac{n_{0}}{{\left(\pi \kappa \theta^{2}\right)}^{3/2}}\frac{1}{U\left(3/2,3/2-\kappa ;\alpha^{2}\kappa \right)}{\left(1+\frac{v^{2}-2e\phi /m_{e}}{\kappa \theta^{2}}\right)}^{-\kappa -1}\times \exp\left(-\alpha^{2}\frac{v^{2}-2e\phi /m_{e}}{\theta^{2}}\right),$$

where *U* (*a*, *b*; *z*) is the Tricomi function (or Kummer *U* function). *κ* and *α* are the superthermal spectral index and the cutoff parameter, respectively, satisfying *κ* > 0 and *α* ≥ 0. $\theta =\sqrt{2T_{e}/m_{e}}$ is the thermal velocity. When we take (a) *α* = 0, Equation (1) reduces to the standard kappa distribution with *κ* > 3/2; when (b) *α* = 0 and κ→∞, Equation (1) reduces to the Maxwellian distribution. Integrating Equation (1) over the velocity space, we can obtain the electron number density *n_e_*

(2) $$n_{e}=n_{e0}\exp\left(\alpha^{2}\frac{e\phi }{T_{e}}\right){\left(1-\frac{e\phi }{\kappa T_{e}}\right)}^{-\kappa +1/2}\frac{U\left(3/2,3/2-\kappa ;\alpha^{2}\kappa \left(1-e\phi /\kappa T_{e}\right)\right)}{U\left(3/2,3/2-\kappa ;\alpha^{2}\kappa \right)}$$

In the case $\left|e\phi /T_{e}\right|<<1$, expanding *ϕ* and retaining the first two terms, we obtain

(3) $$n_{e}=n_{e0}\left(1+\left(\alpha^{2}+\frac{\kappa -\frac{1}{2}}{\kappa }+\frac{3\alpha^{2}U\left(3/2,3/2-\kappa ;\alpha^{2}\kappa \right)}{2U\left(5/2,5/2-\kappa ;\alpha^{2}\kappa \right)}\right)\frac{e\phi }{T_{e}}\right)$$

In the same way, we obtain the ion number density *n_i_*

(4) $$n_{i}=n_{i0}\left(1-\left(\alpha^{2}+\frac{\kappa -\frac{1}{2}}{\kappa }+\frac{3\alpha^{2}U\left(3/2,3/2-\kappa ;\alpha^{2}\kappa \right)}{2U\left(5/2,5/2-\kappa ;\alpha^{2}\kappa \right)}\right)\frac{e\phi }{T_{i}}\right)$$

The Poisson equation for the considered plasma is written as

(5) $$\begin{array}{l} \nabla^{2}\phi =-4\pi e\left(n_{i}-n_{e}\right)\\ =\left(1+\sigma \right)\left(\alpha^{2}+\frac{\kappa -\frac{1}{2}}{\kappa }+\frac{3\alpha^{2}U\left(3/2,3/2-\kappa ;\alpha^{2}\kappa \right)}{2U\left(5/2,5/2-\kappa ;\alpha^{2}\kappa \right)}\right)\left(\frac{4\pi e^{2}n_{0}}{T_{e}}\right)\phi . \end{array}$$

where $\sigma =T_{e}/T_{i}$ is the temperature ratio of electrons to ions. We can easily solve Equation (5), and its solution reads as

(6) $$\phi =\phi_{0}\exp\left(-\frac{x}{\lambda_{D}^{RK}}\right),$$

where the effective Debye shielding length $\lambda_{D}^{RK}$ is given by

(7) $$\lambda_{D}^{RK}=\sqrt{\frac{1}{\left(1+\sigma \right)\left(\alpha^{2}+\frac{\kappa -\frac{1}{2}}{\kappa }+\frac{3\alpha^{2}U\left(3/2,3/2-\kappa ;\alpha^{2}\kappa \right)}{2U\left(5/2,5/2-\kappa ;\alpha^{2}\kappa \right)}\right)}}\lambda_{D},$$

**Figure 1:**
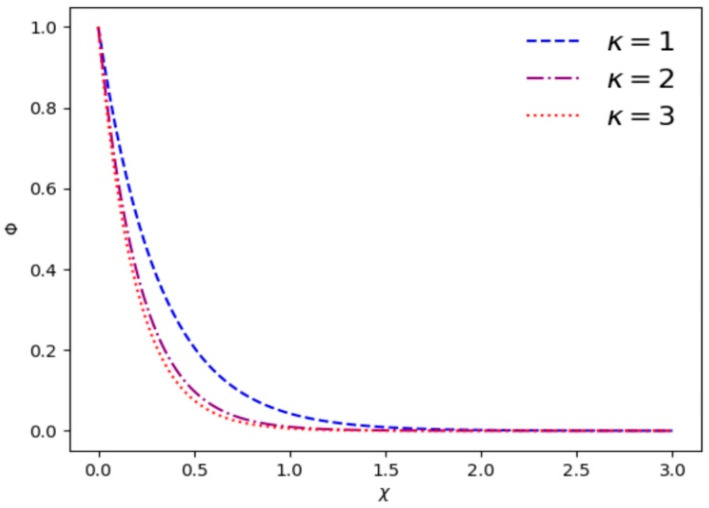
Variation in the electrostatic potential Φ vs. *χ* for different superthermal index *κ* and *α* = 0.1.

**Figure 2:**
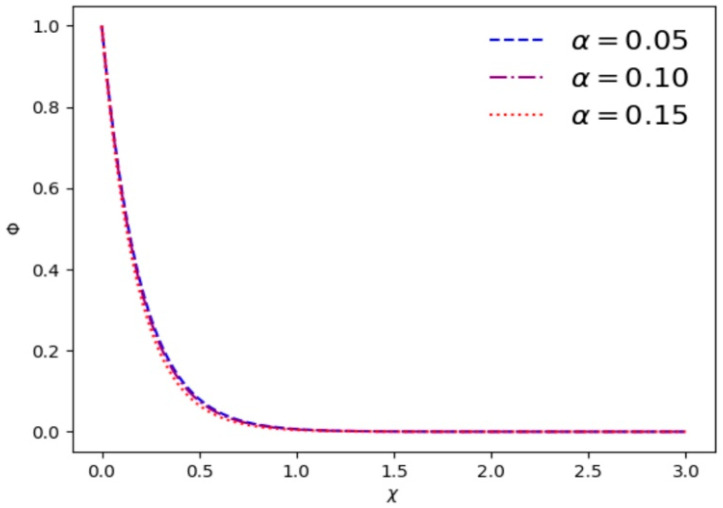
Variation in the electrostatic potential Φ vs. *χ* for different cutoff parameters *α* and *κ* = 3.

Here we note that $\lambda_{D}={\left\{\left(1/1+\sigma \right)\left(T_{e}/4\pi e^{2}n_{0}\right)\right\}}^{1/2}$ is the well-known Debye length for the Maxwellian distributed electron–ion plasma. The effective Debye shielding length $\lambda_{D}^{RK}$ reduces to $\lambda_{D}$ in the limiting case *α* = 0 and κ→∞. Then, we study the effect of *κ* and *α* on the small-amplitude electrostatic potential Equation (6), which can be rewritten as

(8) $$\Phi =\phi /\phi_{0}=\exp\left(-\left(1+\sigma \right)\left(\alpha^{2}+\frac{\kappa -\frac{1}{2}}{\kappa }+\frac{3\alpha^{2}U\left(3/2,3/2-\kappa ;\alpha^{2}\kappa \right)}{2U\left(5/2,5/2-\kappa ;\alpha^{2}\kappa \right)}\right)\chi \right),$$

where $\chi =x/\lambda_{D}$. Figure 1 gives the variation in electrostatic potential Φ as a function of *χ* with different *κ* and *α*. It is found that electrostatic shielding is quite effective beyond a few Debye lengths. Figure 1 shows that the drop of electrostatic potential Φ becomes rapid with the increase in *κ*. Figure 2 exhibits that with increasing values of *α*, electrostatic shielding has a more obvious effect at the same position.

## 3. Electrostatic Sheath

We consider a plasma consisting of cold fluid ions and regularized kappa-distributed electrons, assuming the plasma sheath boundary is located at *x* = 0 (where *ϕ_s_* = 0) with the plasma filling the space *x* < 0, and the plasma sheath is located at *x* > 0. The steady-state continuity and momentum equations for ions in plasma sheath are

(9) $$n_{i}u_{i}=n_{i0}u_{i0},$$

(10) $$\frac{1}{2}m_{i}u_{i}^{2}-\frac{1}{2}m_{i}u_{i0}^{2}=e\phi_{s},$$

where *ϕ_s_* is the electrostatic potential inside the sheath, and *n_i_*_0_ and *v_i_*_0_ are the ion number density and ion drift speed at *x* = 0. From Equations (9) and (10), we can obtain the number density of ions in the electrostatic sheath

(11) $$n_{i}=n_{i0}{\left(1-\frac{2e\phi_{s}}{m_{i}u_{i}^{2}}\right)}^{-1/2}.$$

The Poisson’s equation for the plasma sheath is

(12) $$\frac{\partial^{2}\phi }{\partial x^{2}}=4\pi e\left(n_{e}-n_{i}\right).$$

Then we introduce the following dimensionless variables:

(13) $$\varphi_{s}=-\frac{e\phi_{s}}{T_{e}},\;\xi =\frac{x}{\lambda_{D}}=\frac{x}{\sqrt{T_{e}/4\pi n_{e0}e^{2}}},\;M=\frac{u_{i0}}{C_{s}}=\frac{u_{i0}}{\sqrt{T_{e}/m_{i}}}$$

The Poisson’s Equation (12) can be rewritten as

(14) $$\frac{\partial^{2}\varphi_{s}}{\partial \xi^{2}}=1+\left(\alpha^{2}+\frac{\kappa -\frac{1}{2}}{\kappa }+\frac{3\alpha^{2}U\left(3/2,3/2-\kappa ;\alpha^{2}\kappa \right)}{2U\left(5/2,5/2-\kappa ;\alpha^{2}\kappa \right)}\right)\varphi_{s}-{\left(1-\frac{2\varphi_{s}}{M^{2}}\right)}^{-1/2}.$$

Multiplying both sides of Equation (14) by *dφ_s_*/*dξ*, and integrating it for *ξ* under the boundary conditions (i.e., *φ_s_* = 0, and *dφ_s_/dξ* = 0 at *ξ* = 0), we can obtain the following equation of *φ_s_*

(15) $$\frac{1}{2}{\left(\frac{d\varphi_{s}}{d\xi }\right)}^{2}+V\left(\varphi_{s}\right)=\frac{1}{2}E_{0}^{2},$$

where $E_{0}≃0$ is the weak pre-sheath electric field and

(16) $$V\left(\varphi_{s}\right)=M^{2}\left[1-{\left(1-\frac{2\varphi_{s}}{M^{2}}\right)}^{1/2}\right]-\varphi_{s}-\frac{1}{2}\left(\alpha^{2}+\frac{\kappa -\frac{1}{2}}{\kappa }+\frac{3\alpha^{2}U\left(3/2,3/2-\kappa ;\alpha^{2}\kappa \right)}{2U\left(5/2,5/2-\kappa ;\alpha^{2}\kappa \right)}\right)\varphi_{s}^{2}$$

is the Sagdeev potential. It is easily seen that the solution of Equation (15) exists if $d^{2}V\left(\varphi_{s}=0\right)/d\varphi_{s}^{2}<0$ or

(17) $$M>M_{c}=\sqrt{\frac{1}{\alpha^{2}+\frac{\kappa -\frac{1}{2}}{\kappa }+\frac{3\alpha^{2}U\left(3/2,3/2-\kappa ;\alpha^{2}\kappa \right)}{2U\left(5/2,5/2-\kappa ;\alpha^{2}\kappa \right)}}}$$

Equation (17) is the modified Bohm sheath criterion in an electron–ion plasma with regularized kappa distributed electrons. In the limiting condition *α* = 0 and κ→∞, Equation (17) becomes *M* > 1 for Maxwellian cases.

**Figure 3:**
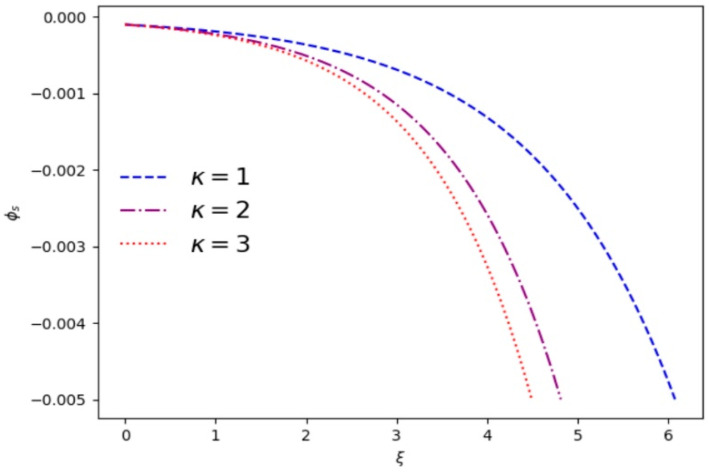
Variation in the shielded electrostatic potential *ϕ_s_* vs. *ξ* for different superthermal index *κ* and *α* = 0.1.

**Figure 4:**
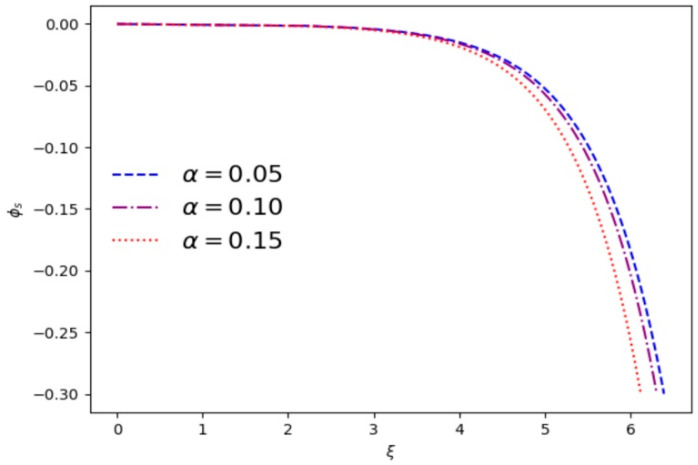
Variation in the shielded electrostatic potential *ϕ_s_* vs. *ξ* for different cutoff parameters *α* and *κ* = 3.

Figure 3 and Figure 4 numerically show the variation in the shielded electrostatic potential *ϕ_s_* with distance for different plasma parameters. The sheath region begins where charge neutrality breaks down. Figure 3 shows that by increasing *κ*, the fall-off of *ϕ_s_* through the sheath region becomes more rapid and the thickness of sheath decreases. For the larger *κ*, the average velocity of electrons is relatively slower, which may affect the electrostatic potential. Figure 4 shows that the electrostatic potential *ϕ_s_* and the thickness of sheath decrease with the increase in *α*.

## 4. Conclusions

In this paper, we have examined the phenomenon of Debye shielding in an electron–ion plasma with regularized kappa distribution. The unmagnetized collisionless plasma sheath with regularized kappa distributed electrons is investigated and the modified Bohm criterion is derived. It is found that the variation in the electrostatic potential depends significantly on the superthermal index *κ* and cutoff parameters *α*. The electrostatic potential *ϕ_s_* falls more quickly with the increase in *κ* and *α*. The thickness of sheath becomes thicker for the smaller values of *κ* and *α*. It is worth noting that the plasma sheath exists with the regularized kappa distribution if *κ* < 3/2. The presence of regularized kappa-distributed electrons has been observed in the Earth’s inner magnetosphere [33]. Our present work may be useful in understanding plasma sheath and plasma processing in related plasma regions.

## Data Availability

The data presented in this study are available on request from the corresponding author.
